# Acute Left Lower Limb Swelling in an Elderly Patient

**DOI:** 10.1016/j.acepjo.2025.100219

**Published:** 2025-07-16

**Authors:** Tlaite Oubaddi, Meriem Zhim, Mohammed Lahkim, Jamal El Fenni

**Affiliations:** Department of Radiology, Mohammed V Military Teaching Hospital, Mohammed V University, Rabat, Morocco

**Keywords:** CT angiography, arteriovenous fistula, iliac aneurysm, vascular surgery

## 1 Case Presentation

An 87-year-old man, with a history of hypertension and total right hip prosthesis, presented with a 1-day history of sudden-onset swelling of the left lower limb. Examination revealed marked swelling and cyanosis of the limb, with symmetrical and palpable distal pulses. He was hemodynamically stable, with a blood pressure of 98/60 mmHg and a heart rate of 85 bpm.

## 2 Diagnosis: Ruptured Iliac Aneurysm Resulting in Ilio-Iliac Arteriovenous Fistula

**Figure 1:**
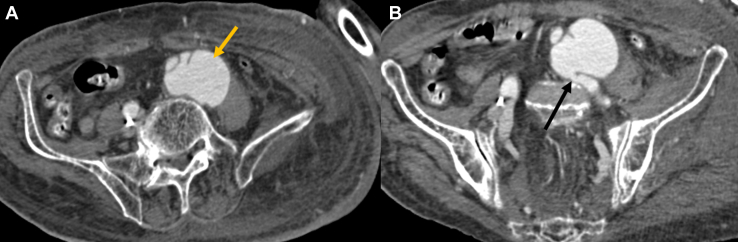
A CT angiography revealed a saccular aneurysm of the left common iliac artery (yellow arrow), with a fistulous communication to the left common iliac vein (black arrow). CT, computed tomography.

**Figure 2:**
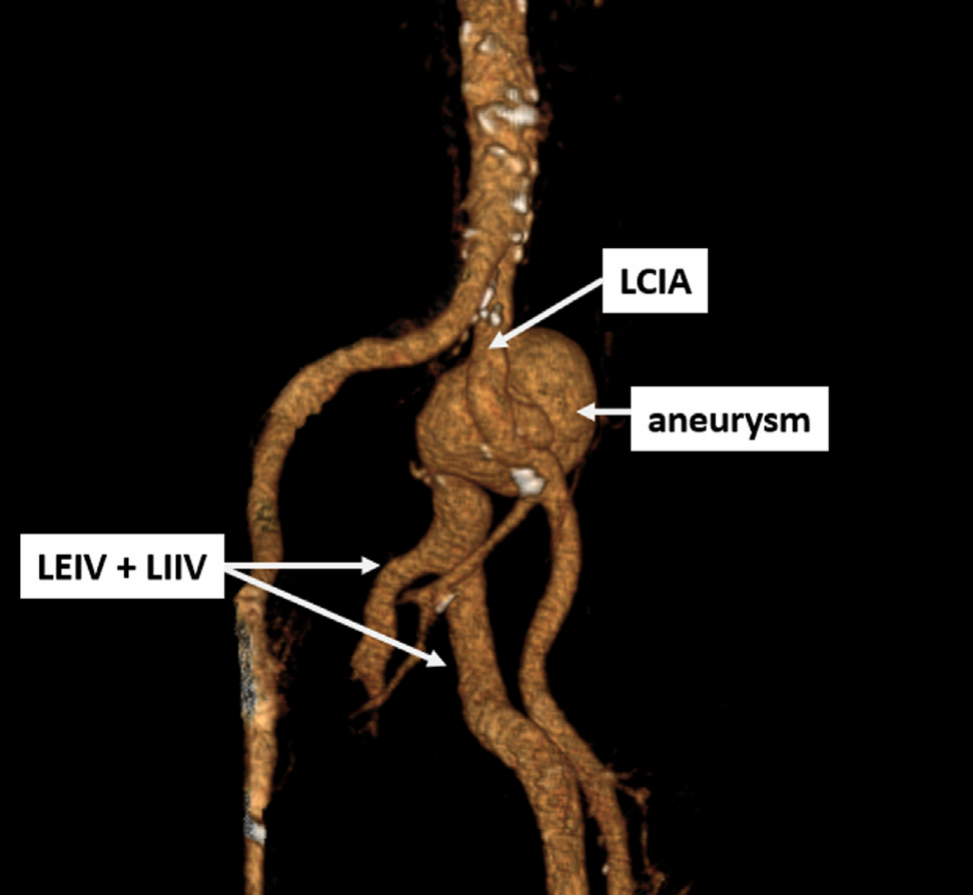
A 3D reconstruction demonstrated an aneurysm of the LCIA, with arterial opacification of the left external and internal iliac veins (LEIV and LIIV). 3D, three-dimension. LCIA, left common iliac artery; LEIV, left external iliac vein; LIIV, left internal iliac vein.

**Figure 3:**
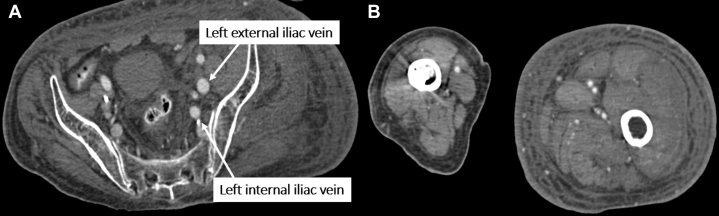
(A) CT angiography demonstrated arterial opacification of the left iliac veins (arrows), associated with swelling of the left lower limb (B). CT, computed tomography.

Computed tomography (CT) angiography revealed a saccular aneurysm of the left common iliac artery (Figs 1A and 2) with a fistulous communication to the left common iliac vein (Fig 1B), causing early opacification of the left iliac veins in the arterial phase (Fig 3), consistent with an acquired ilio-iliac arteriovenous fistula secondary to aneurysmal fistulization. The patient underwent emergent open repair with fistula closure and iliac artery graft replacement.

Ilio-iliac arteriovenous fistulas caused by iliac aneurysms are rare but can cause significant hemodynamic instability. Only one-third of patients present with the classic triad of a pulsatile abdominal mass, high-output cardiac failure, and unilateral limb ischemia or venous engorgement. CT angiography is the diagnostic gold standard showing dilation of the draining vein and arterialization of the iliac vein and vena cava. Definitive treatment is surgical, involving fistula closure and vascular reconstruction, typically with a bifurcated aortic graft.^1^

## 3 Teaching Points

Ilio-iliac arteriovenous fistulas are rare but serious complications of iliac aneurysms, requiring prompt CT diagnosis and surgical management.

## Conflict of Interest

All authors have affirmed they have no conflicts of interest to declare.
